# Obituary

**Published:** 1903-03

**Authors:** 


### Obituary.

Thomas John Colman, M.D. Edin. 1868, M.R.C.S. Eng. 1864, L.S.A. 1865, whose decease took place in London on January 2nd, at the age of 60, will be long mourned by a large circle of medical, musical, and lay friends. He was a native of Bristol, and was a student at the Bristol Medical School; he afterwards went to Edinburgh, and became House Physician to the Royal Infirmary and Assistant-Physician at the Royal Edinburgh Asylum, Morningside. Before commencing private practice, he became House Surgeon at the Bristol General Hospital, and, later, he served for two years as Surgeon to the Bristol Royal Hospital for Sick Children and Women.

During the early years of his professional life he spent much of his time in the cultivation of his musical tastes. As a result of steady perseverance and long practice, his knowledge of music became both varied and wide, and his power of execution was of a very high order. He was much in demand at social functions, and his musical evenings were always very enjoyable. In spite of all this, he did not neglect his professional work, and his practice in all parts of Bristol grew rapidly to dimensions which were beyond the control and physical capacity of one who was never robust, and who found the fatigue entailed by incessant work to be beyond his strength. In 1887 Dr. Leonard Lees shared the labours of his practice, and continued to work with him for twelve years.

For several years past he had been frequently obliged to obtain surgical assistance, and had been doing his work under great difficulty. He was much beloved by his patients, and his funeral, on January 7th, was attended by a very large congregation of friends and patients, when the floral display gave evidence of a very widespread sympathy with his relatives in their loss.

Those who had the privilege of knowing him personally marvelled at his untiring energy, his high sense of duty, and devotion to his work. He endeared himself to his patients by his great sympathy and kindness, frequently performing acts for them, in order to relieve suffering, not merely medical, but those of a nurse, and thus earning for himself the eternal gratitude of many who came under his care. He was a man of large experience, with a nature retiring and sensitive in the extreme; strong in his likes and dislikes, a true friend to those who gained his confidence. His was a busy life, and his tastes were of the simplest character; he was the soul of honour, and a man of the highest principles. His influence for good was felt by all who were intimately associated with him, and who now mourn his loss; and he set an example which it is well to follow.

